# Engaging Nigerian community pharmacists in public health programs: assessment of their knowledge, attitude and practice in Enugu metropolis

**DOI:** 10.1186/s40545-015-0048-0

**Published:** 2015-11-09

**Authors:** Ogochukwu Offu, Maureen Anetoh, Matthew Okonta, Obinna Ekwunife

**Affiliations:** Department of Pharmacy, Enugu State University Teaching Hospital, Enugu, Nigeria; Department of Clinical Pharmacy and Pharmacy Management, Nnamdi Azikiwe University, Awka, Nigeria; Department of Clinical Pharmacy and Pharmacy Management, University of Nigeria, Nsukka, Nigeria; Collaborative Research Group for Evidence-Based Public Health, Department of Prevention and Evaluation, Leibniz Institute for Prevention Research and Epidemiology – BIPS/University Bremen, Bremen, Germany

**Keywords:** Community pharmacy, Health promotion, Disease prevention, Nigeria

## Abstract

**Objectives:**

The Nigerian health sector battles with control of infectious diseases and emerging non-communicable diseases. Number of healthcare personnel involved in public health programs need to be boosted to contain the health challenges of the country. Therefore, it is important to assess whether community pharmacists in Nigeria could be engaged in the promotion and delivery of various public health interventions. This study aimed to assess level of knowledge, attitude and practice of public health by community pharmacists.

**Methods:**

The cross sectional survey was carried out in Enugu metropolis. Questionnaire items were developed from expert literature. Percentage satisfactory knowledge and practice were obtained by determining the percentage of community pharmacists that were able to list more than 2 activities or that stated the correct answer. Attitude score represents the average score on the 5 point Likert scale for each item. Chi square and Fisher’s exact test were used to test for statistically significant difference in knowledge, attitude and practice of public health between different groups of community pharmacists.

**Results:**

Forty pharmacists participated in the survey. About one third of the participants had satisfactory knowledge of public health. With the exception of one item in attitude assessment, average item score ranged from ‘agreed’ to ‘strongly agreed’. Study participants scored below satisfactory on practice of public health. Knowledge, attitude and practice of public health were not influenced by years of practice, qualification and prior public health experience. Reported barriers to the practice of public health include inadequate funds, lack of time, lack of space, cooperation of clients, inadequate staff, government regulation, insufficient knowledge, and remuneration.

**Conclusions:**

Level of knowledge and practice of public health by community pharmacists were not satisfactory although they had a positive attitude towards practice of public health. The findings highlight the importance of educational interventions targeted towards practicing community pharmacists to improve their knowledge level on public health issues. Providing incentives for public health services rendered could increase community pharmacists’ engagement in public health activities.

## Introduction

Nigeria has poor health indices which are largely due to inadequate health infrastructure and services that could address the numerous health challenges. Overall life expectancy at birth is 54 years while maternal mortality ratio is 560 per 100,000 live births [1]. The Nigerian health sector battles with control of infectious diseases as well as the rapid and on-going emergence of non-communicable diseases. Studies have indicated rising prevalence of hypertension, diabetes mellitus and obesity in the country [2–4]. Among the many challenges of public health in Nigeria is the low number of adequately trained healthcare workforce. Densities of nurses, midwives and doctors (1.95 per 1000) are still considered low for effective delivery of essential health services [5].

The community pharmacist has been an important health resource that could be engaged in promoting and delivering various public health interventions. Accessibility of the community pharmacists makes them a good means of delivering public health interventions. In recent times, the role of community pharmacists is expanded to include public health services [6]. The new community pharmacist role in public health could be better understood when viewed from 3 levels on preventions: primary, secondary, tertiary [6]. Primary prevention involves intervening to inhibit the initiation of a negative health outcome such as pharmacist involvement in distribution of vaccine materials and latterly provision of immunization in some countries. Secondary prevention involves intervening early in the disease process and before illness manifests. Examples include early intervention for behavior change or disease diagnosis in cases of cardiovascular disease, diabetes, substance abuse etc. Tertiary prevention activities seek to slow the progression of disease and reduce complications through medication evaluations especially for special group of patients e.g., HIV patients, patients on multiple and possibly conflicting medications due to cormobidities etc.

The Pharmacist Council of Nigeria (PCN) which is a body in charge of regulation of pharmacy practice in Nigeria recognizes this changing role of the community pharmacist [7] and has formulated new practice standards to assure practice of pharmaceutical care [8]. The standards explicitly stipulate a good number of public health activities. These standards include health promotion activities on smoking cessation, nutrition, HIV/AIDS, infectious disease control, family planning, control of sexually transmitted diseases (STDs), baby friendly initiative programs, drug use in pregnancy, rational use of drugs, and substance abuse, The standards also stipulate disease prevention activities such as immunization campaign and screening for chronic disease.

To ensure adherence to stipulated guideline and hence improvement of public health practice in community pharmacies in Nigeria, it is pertinent to assess the knowledge base of pharmacy practitioners with regards to public health and characterize their current level of public health practice as well as their attitude toward carrying out public health interventions in their premises. Other studies in Nigeria have assessed attitudes of community pharmacists towards health promotion [9] and their involvement in primary healthcare [10, 11]. Our study specifically characterizes the knowledge base, attitude and level of practice of public health by Nigerian community pharmacists. Specifically, the study aims to: (i) assess knowledge, attitude and practice of public health by community pharmacists; (ii) and determine barriers that prevent the community pharmacists from practicing public health activities.

## Method

The cross sectional survey was carried out in Enugu metropolis, in south eastern part of Nigeria. Enugu has an estimated population of 3,257,298 [12]. There were 98 registered community pharmacies in the state as at January 2015. Enugu was purposively selected since different cadres of pharmacies operate in the city. For instance, some of the biggest pharmacy chains in Nigeria (HealthPlus and Mopheth®) as well as small individually owned pharmacies operate in Enugu.

Criteria for inclusion were registration with Pharmacist Council of Nigeria (PCN) and a full or part time pharmacist(s) working in the community pharmacy. With a population of 98 pharmacies and assuming a confidence level of 95 % with a confidence interval of ±12, a sample size of 40 pharmacies was estimated to be adequate for the survey [13]. Stratified sampling technique was employed to select pharmacies for the study. Enugu City was divided into ten strata namely: Trans Ekulu, Abakpa, New Haven, Coal Camp, Independence Layout, Ogui, Achara layout, Uwani, GRA (Government Residential Area), and Ogbete. Four community pharmacies were randomly selected from each stratum.

Questionnaire items were developed after reviewing the PCN guideline [8], International Federation of Pharmacy (FIP) policy statements [14], and other publications on the role of community pharmacists in public health [15, 16]. The questionnaire was made up of four sections: demographics, knowledge, attitude and practice. Questions in the knowledge and practice sections were open ended. For the attitude section, a 5-point Likert scale with 5 as “strongly agreed” and 1 as “strongly disagreed” was used. Some items were worded negatively to avoid response bias. The negatively worded questions were reversed at the point of analysis. A midpoint of 3 represented neutral point between poor attitude and good attitude. The questionnaire was face validated by two pharmacists (an experienced community pharmacist and an academic pharmacist) and was pilot tested using 10 pharmacists (not in community practice) to assess feasibility and possible comprehension problems. Construct validity was assessed by choosing two pairs of items which were subjectively judged by the researchers not to be related. However, the items of each pair were judged to be related and dependent on each other [17]. Convergent and discriminant validities of these items were then computed to determine the validity of the instrument’s construct. Bivariate correlation with Pearson coefficient was used to establish convergence and discrimination. Modifications were made to the questionnaire based on problems identified during the feasibility study. The final questionnaire was distributed to the 40 selected pharmacies in Enugu.

The study was approved by the local ethics committee of the Faculty of Pharmaceutical Sciences, Enugu State Ministry of Health. All study participants were given prior information on the nature of study. Oral informed consent was obtained. Anonymity and confidentiality of participants were respected by the researchers. The questionnaires were self administered. Data collection was carried out for 6 months from October 2014 to March 2015.

Data was coded and entered into SPSS version 14 (Chicago, IL). Open ended questions were thematically coded and matched against standard definitions or activities previously agreed upon before analysis. Percentage satisfactory knowledge and practice was obtained by determining the percentage of community pharmacists that were able to list more than 2 activities or that stated the correct answer. Attitude score represents the average score on the 5 point Likert scale for each item. Chi square or Fisher’s exact test were used to test for statistically significant difference in knowledge, attitude and practice of public health between different groups of community pharmacists. The groups considered were young graduates (1–5 years) vs old graduates (>5 years); graduate degree vs postgraduate degree; public health experience vs no public health experience. A two tailed significance value of 0.005 was used.

## Results

The two pairs of items used to determine the validity of construct were questions 1 and 2 and questions 4 and 10 from the attitude assessment section (See Table 3). Correlation values ranging from 0 to 0.5 were set to indicate divergent validity while values ranging from 0.5 to 1.0 would indicate convergent validity. Question 1 versus question 2 had a correlation value of 0.598 (*p* < 0.001) while items 4 and 10 had a value of 0.350 (*p* < 0.05) showing that the first pair had a convergence and the latter pair had a slight convergence. The correlation values of questions 1 and 4, 1 and 10, 2 and 4, and 2 and 10 were 0.291 (*p* = 0.069), 0.236 (*p* = 0.143), 0.223 (*p* = 0.167) and 0.116 (*p* = 0.478) respectively. These values signify discriminant validity, showing that the items from the different scales were significantly different and were independent of each other.

None of the community pharmacists approached declined to participate. Majority of community pharmacists were males and with first degree (Bachelor of Pharmacy). About one half of the participants were between the ages of 31–40 years. Thirty five percent of the study participants had previously worked in a public health setting. Details of demographic characteristics of the study participants are shown in Table 1.

**Table 1:** Demographic characteristics of respondents

Characteristics		Frequency (%)
Sex	Male	33 (82.5)
	Female	7 (17.5)
Age (years)	21–30	14 (35.0)
	31–40	19 (47.5)
	41–50	5 (12.5)
	51–60	1 (2.5)
	>60	1 (2.5)
Years of Practice	1–5 years (Young graduates)	22 (55.0)
	>5 years (Old graduates)	18 (45.0)
Qualification of pharmacist	Graduate degree	30 (75.0)
	Postgraduate degree	10 (25.0)
Previously worked in Public Health Setting	No	26 (65.0)
	Yes	14 (35.0)

Item analysis of community pharmacists’ knowledge of public health is show in Table 2. With reference to CEA Winslow’s definition [18], only 10 % of the pharmacists could correctly define public health. About 20 % listed correctly three or more health promotion activities that could be conducted in community pharmacies. Most of the pharmacists could not explain disease prevention in the context of public health. However, one-half of them were able to state more than two disease prevention activities. On the average, about one third of the participants had satisfactory knowledge of public health. Knowledge of public health was not influenced by years of practice, qualification and prior public health experience.

**Table 2:** Item analysis of community pharmacists’ knowledge of public health

	Item	Satisfactory knowledge (%)^a^	*P*-values		
			Years of practice	Qualification	Public health experience
1	What is public health in your own words?	10.0	0.29	0.21	0.32
2	What is health promotion?	27.5	0.23	0.32	0.19
3	List three health promotion activities that the community pharmacist can carry out?	22.5	0.52	0.90	0.75
4	State means through which health education can be given.	30.8	0.14	0.98	0.64
5	What is disease prevention?	3.5	0.56	0.25	0.32
6	State examples of disease prevention activities that the community pharmacist can carry out.	50.0	0.14	0.08	0.20
7	How does a community pharmacist contribute to prolonging the life of a client who has a chronic disease?	80.0	0.71	0.65	1.00
	Average satisfactory knowledge score	31.9	-	-	-

^a^Listed >2 activities/stated the correct answer

With regards to attitude towards public health, the pharmacists had a good attitude toward practice of public health. With the exception of item 4, average item scores ranged from ‘agreed’ to ‘strongly agreed’. The total attitude score was above the neutral point of 36. Attitude towards public health was not influenced by years of practice, qualification and prior public health experience. Details are shown in Table 3.

**Table 3:** Item analysis for community pharmacists’ attitude to public health

Item		Mean ± SD	*p*-values		
			Years of practice	Qualification	Public health experience
^a^1.	Public Health activities should be carried out by only nurses and medical doctors.	4.7 ± 0.7	0.56	0.71	0.74
^a^2	Public Health activities should be carried out in health centres only.	4.5 ± 0.7	0.64	0.81	0.47
^a^3.	Pharmacists were not trained to carry out public health activities while in pharmacy school.	4.3 ± 0.9	0.71	0.70	0.28
4.	Community pharmacists are competent to carry out public health activities.	4.3 ± 1.0	0.23	0.57	0.22
5.	Health education carried out by the community pharmacist should be focused on group of individuals as well as individual clients	3.9 ± 1.1	0.06	0.19	0.27
^a^6.	Health education administered by community pharmacists should focus on drug related information.	4.2 ± 0.7	0.84	0.74	0.84
^a^7.	It is not the role of community pharmacists to educate their clients on risk factors to chronic diseases, such as smoking, nutrition, overweight, etc.	4.8 ± 0.4	0.68	0.34	0.39
^a^8.	It is not essential for the community pharmacists to educate their clients on oral hygiene/health.	4.7 ± 0.7	0.65	0.43	0.69
^a^9.	It is not necessary for the community pharmacists to educate their clients on sexual health (contraception, prevention of STIs) since it is already done in the family planning clinic.	4.6 ± 0.5	1.00	0.48	0.33
^a^10.	Educating clients on immunization (including vaccines required for intending travelers) should not be the business of community pharmacists.	4.6 ± 0.7	0.53	0.76	0.23
11.	Screening clients in the community pharmacy for hypertension, diabetes and dyslipidemia will help to reduce the incidence and prevalence of cardiovascular diseases.	4.7 ± 0.8	0.46	0.17	0.71
12.	It is important for the community pharmacists to always ensure that patients adhere to their medication.	4.7 ± 0.7	0.39	0.18	0.70
	Average attitude score	4.5 ± 0.7	-	-	-

^a^A high score for these items were reversed to the corresponding low score and vice versa at the point of analysis

Table 4 shows the item analysis of community pharmacist’s practice of public health. Majority of the pharmacists screen for hypertension and diabetes while about a quarter monitor drug abuse/ensure safe use of drugs and conduct weight checks (Table 5). As shown in Table 4, only about 28 % could list three or more health issues that they educate their patients about. Fifteen percent of the pharmacists could list three or more diseases that they screened for in their pharmacies. On the average, satisfactory practice score was low. Practice of public health was not influenced by years of practice, qualification and prior public health experience.

**Table 4:** Item analysis of community pharmacist’s practice of public health

Number	Activities	Satisfactory practice (%)^a^	*p*-value		
			Years of practice	Qualification	Public health experience
1.	List five public health activities that you carry out in your pharmacy?	87.5	0.44	0.39	0.68
2.	Name the health promotion activities you are involved in?	27.5	0.13	0.85	0.36
3.	What issues do you educate your patients about?	27.5	0.20	0.30	0.86
4	List the diseases you screen for in your pharmacy	15.0	0.30	0.21	0.73
	Average satisfactory practice score	39.4	-	-	-

^a^Listed >2 activities/stated the correct answer

**Table 5:** Public health activities indicated to be carried out in community pharmacies

Activities	Frequency	% involvement
Screening for diabetes	27	67.5
Screening for hypertension	27	67.5
Drug abuse/safe use of drugs	11	27.5
Weight checks	11	27.5
General/personal hygiene	9	22.5
Advice and treatment of sexually transmitted infection/HIV/AIDS	6	15
Family planning/emergency contraception	4	10
Advice on smoking cessation	3	7.5
Education on physical activity	3	7.5
Screening for dyslipidemia	3	7.5
Management of common ailments	3	7.5
Awareness of immunization/vaccination	2	5
Breast feeding	2	5
Screening for malaria	2	5
Body mass index (BMI) measurement	2	5
Breast feeding	2	5
Ovulation/pregnancy test	1	2.5
Folic acid supplementation for child bearing women	1	2.5

**Fig. 1:**
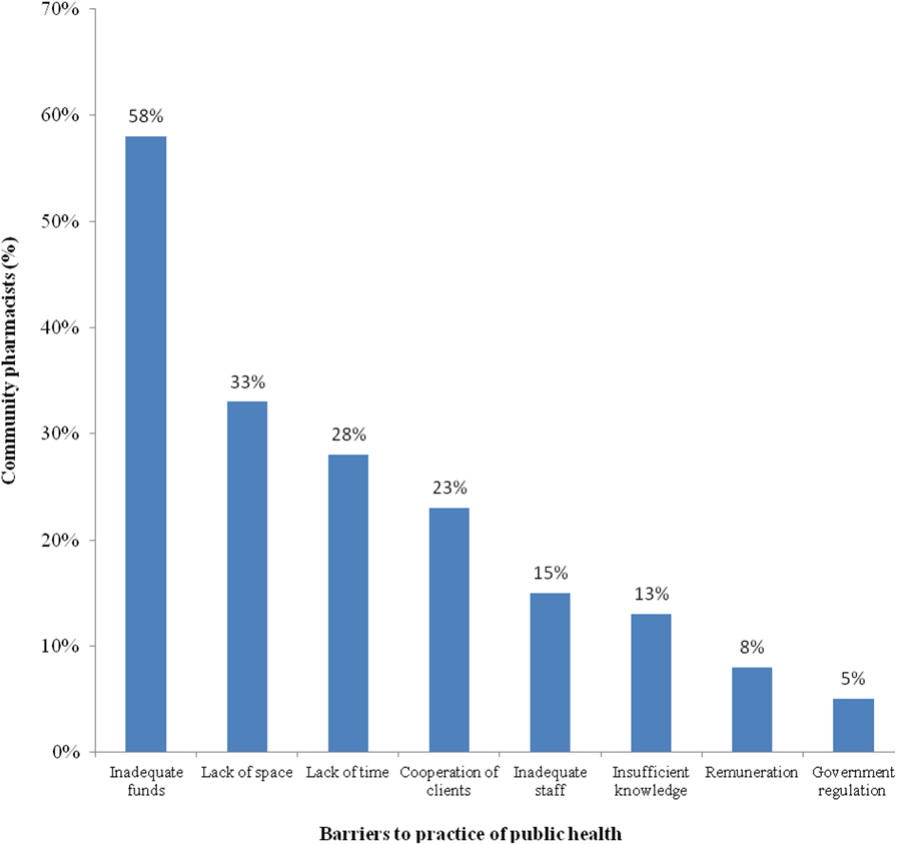
Reported barriers affecting practice of public health in community pharmacy

Perceived barriers affecting public health practice in community pharmacy are shown in Fig. 1. More than half of the community pharmacists reported inadequate funds as barrier to the offering of public health services. Other reported barriers were lack of space, lack of time, cooperation of clients and inadequate staff.

## Discussion

### Knowledge, attitude and practice of public health

This study showed that although community pharmacists had a positive attitude towards the practice of public health, their knowledge and practice level was poor. The findings did not differ among community pharmacists with higher number of years of practice, higher qualification or those with prior public health work experience. Positive attitude of community pharmacists observed in this study is an indication that community pharmacists may be willing to offer public health services. Our finding is similar to another Nigerian based survey which showed that 84 % of Nigerian community pharmacists indicated a favorable attitude towards health promotion [9]. Similar findings of positive attitude of community pharmacists towards practice of public health have been reported in other developing country [19] and developed countries [15, 20].

### Educational interventions for better public health practice

Poor knowledge in public health issues as evidenced by the results of this study could have resulted in low level of engagement in public health activities. Our findings highlight the importance of educational interventions targeted towards practicing pharmacists to improve their knowledge level on public health issues. In a similar study conducted elsewhere in Nigeria, many community pharmacists agreed that their participation in primary healthcare could be further improved through continuous education and training [10]. This may not be peculiar to Nigeria as a UK based study indicated that training and support is needed in order to increase pharmacist’s confidence in providing public health services [15]. As a suggestion, training in public health could be delivered through the mandatory continuing professional development (MCPD) organized by the Pharmacists Council of Nigeria (PCN), as pharmacists of greater than 5 years in practice are mandated to undertake update courses as part of their licensure requirement. Another cost-effective initiative could be an online learning platform for practicing community pharmacists on public health topics. To motivate pharmacists to take the e-courses, the courses could be accredited by PCN as part of the fulfillment for licensure requirement.

### Reported barriers: need for incentive

Perceived barriers to public health practice in the community pharmacy according to the respondents were inadequate funds, inadequate staff, government regulation, insufficient knowledge, lack of time, lack of space, and cooperation of clients. These perceived barriers are not peculiar to the Nigerian scenario as they have been reported in other studies [15, 19]. In our opinion, these reported barriers are connected to lack of incentive. Most community pharmacists would be interested in making sales to improve business profitability rather than offering free public health services. They may not consider it necessary to find solutions to the reported barriers if not properly incentivized. Pharmacy in Nigeria is not yet formally classified as a profession within the public health workforce and also the public health role of the pharmacist is yet to be sufficiently recognized and promoted by public health agencies, pharmacy educators, or other healthcare professionals. This may explain why community pharmacists are not officially part of public health programs and thus are not reimbursed for services offered.

### Strengthening pharmacists as public health partners

Community pharmacists could be positioned to promote and deliver various public health interventions. Unfortunately lack of preparedness results in missed opportunities to intervene in both infectious and non infectious diseases plaguing the country. Our findings showed that screening for diabetes and hypertension were the public health activities mostly carried out in community pharmacies. Community pharmacists could be useful in screening and detection of other disease states. For instance, to boost the number of health workers involved in provision of maternal, newborn and child health (MNCH) in the country, community pharmacists could be positioned as promoters, facilitators and implementers of maternal, newborn and child health [21]. Community pharmacists are easily accessible in the community and are often the first point of call for majority of Nigerians. A baseline study of the community pharmacists’ participation in MNCH in Nigeria revealed a considerable client load of pregnant and nursing mothers with children under 5 years in contact with the community pharmacists daily [22]. Specifically, over 15 % of community pharmacists see between 5 and 10 pregnant women and 10–20 children per day [22].

Strengthening pharmacists as public health partners will require combined effort of pharmacy practice administrators, academic pharmacists and practicing pharmacists to evolve policies supported by evidence showing positive impact of pharmacy-based public health activities. This will aid pharmacists to be formally classified as professionals within the public health workforce and their role in public health recognized by public health agencies. Strengthening pharmacists as public health partners will also require meaningful integration of pharmacy and public health in practice and education. Example of such integration include dual-degree programs and integrated curricula which offers rich avenues for thoughtful integration, local departments and pharmacies partnering to provide HIV testing for instance, educational integration by appointing public health faculty to schools of pharmacy and vice versa etc. [6].

### Study limitations

One major limitation to be considered while interpreting the result of the findings is the generalizability of the result. However, as stated earlier, and effort was made to choose a city which presents a fair representation of community pharmacy practice in Nigeria as all the cadres of community pharmacies in terms of size operates in Enugu. Also, the small sample size of the community pharmacists studied may have not permitted detection of significant differences where expected. For instance, prior experience of public health showed no significant effect on the public health knowledge of the community pharmacists.

## Conclusion

Using Enugu city as a case study, knowledge and practice of public health by Nigerian community pharmacists was not satisfactory although they had a positive attitude towards practice of public health. The findings highlight the importance of educational interventions targeted towards practicing community pharmacists to improve their knowledge level on public health issues. Providing incentives for public health services rendered could increase community pharmacists’ engagement in public health activities.

## Abbreviations

PCN: Pharmacists council of Nigeria
GRA: Government residential area
FIP: Federation of pharmacy
STDs: Sexually transmitted diseases
MCPD: Mandatory continuing professional development
MNCH: Maternal, newborn and child health
